# Angiomatous Urethral Caruncle After Ureteroscopy: A Case Report

**DOI:** 10.7759/cureus.47528

**Published:** 2023-10-23

**Authors:** Hicham El Boté, Jihad Lakssir, Abdelmounim Boughaleb, Omar Bellouki

**Affiliations:** 1 Surgery, Faculty of Medicine and Pharmacy of Beni Mellal, Regional Hospital of Beni Mellal, Beni Mellal, MAR; 2 Urology, Ibn Sina Hospital, University of Rabat, Rabat, MAR

**Keywords:** urethra lesions, urethral meatus, angiomatous caruncle, ureteroscopy, urethral caruncle

## Abstract

Urethral caruncle is a rare condition. Although relatively common in postmenopausal women, its occurrence after a urological endoscopic procedure is unusual. Here, we report the case of a postmenopausal woman who presented with a symptomatic urethral caruncle two weeks after a ureteroscopy for a right ureteral calculus. Treatment consisted of surgical excision of the mass after the failure of local estrogen application, and the postoperative aesthetic and functional result was satisfactory. Through a review of the literature, the etiological, diagnostic, and therapeutic aspects of this pathology will be discussed.

## Introduction

Urethral caruncle is a benign lesion with a significantly high incidence in postmenopausal women [1]; however, its occurrence after urologic endoscopic procedures such as ureteroscopy is unusual.

Here, we report the case of a postmenopausal woman who presented with a symptomatic urethral caruncle two weeks after a ureteroscopy for right ureteral calculus. The underlying etiologies, diagnosis, and treatment of this uncommon condition are briefly discussed.

## Case presentation

A 63-year-old postmenopausal woman with a history of bilateral deafness was treated with cochlear prostheses. She presented with hyperalgesic right renal colic due to an obstructive 10 mm pelvic ureteral calculus. She underwent minimally invasive semi-rigid ureteroscopy with complete fragmentation of the stone using the holmium laser.

**Figure 1:**
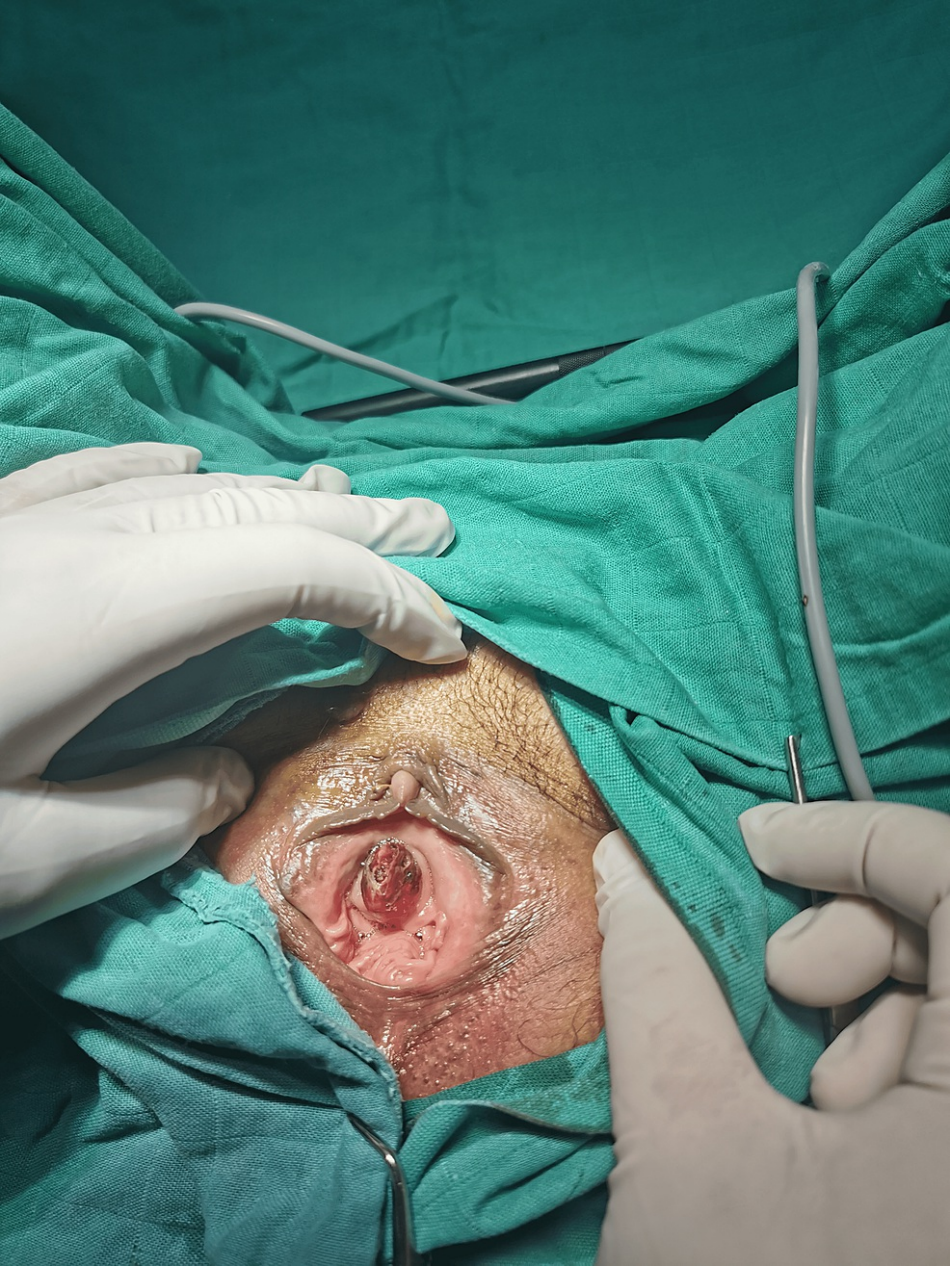
Preoperative appearance of the urethral caruncle.

**Figure 2:**
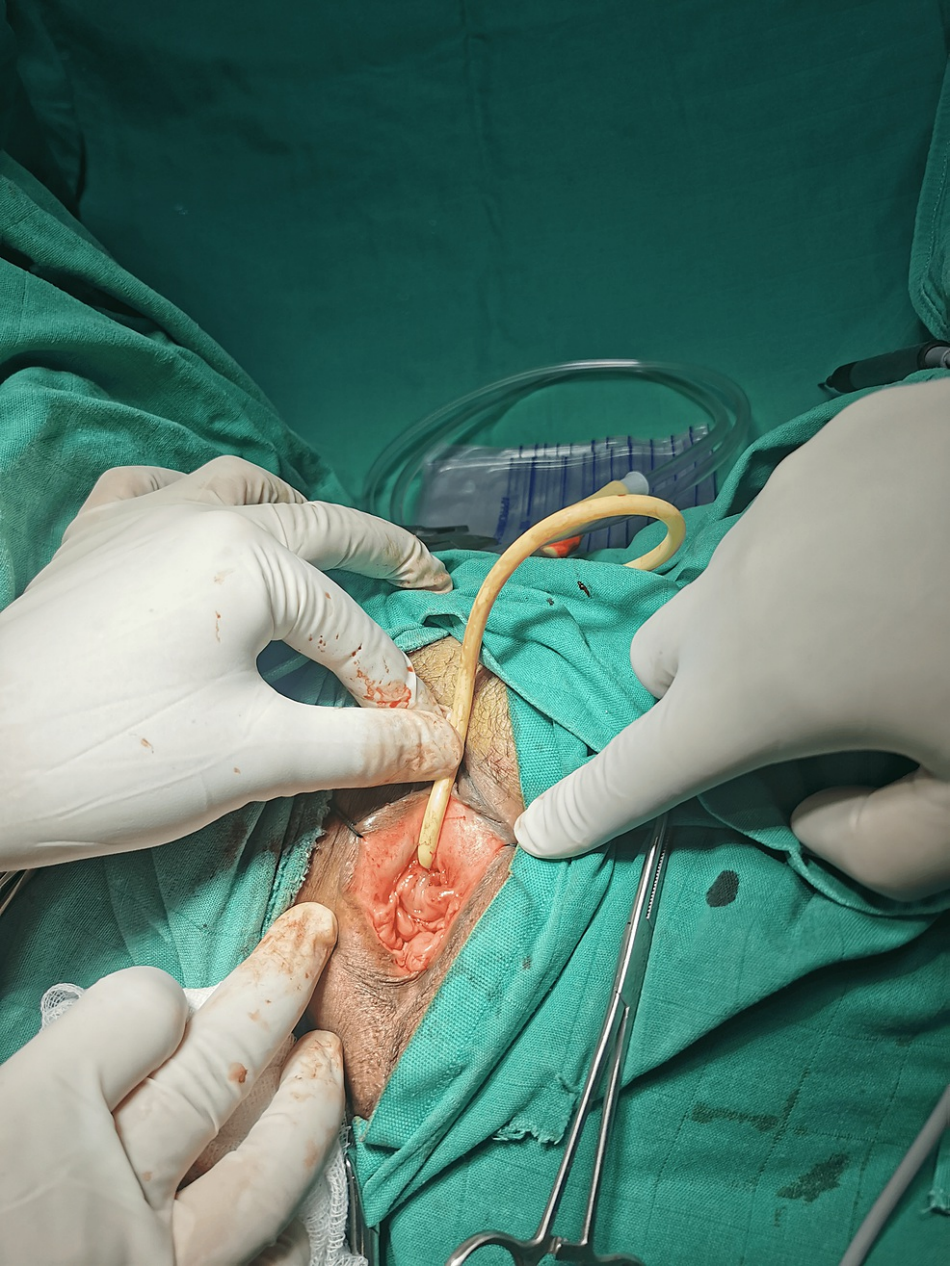
Postoperative appearance after the removal of the urethral caruncle.

Two weeks later, she presented with intermittent bleeding and dysuria. Clinical examination revealed a 2.5 cm bulging mass externalized through the urethral meatus, bleeding on contact (Figure 1). After the failure of local estrogen treatment, total surgical removal of the mass with a Foley catheter was performed (Figure 2).

**Figure 3:**
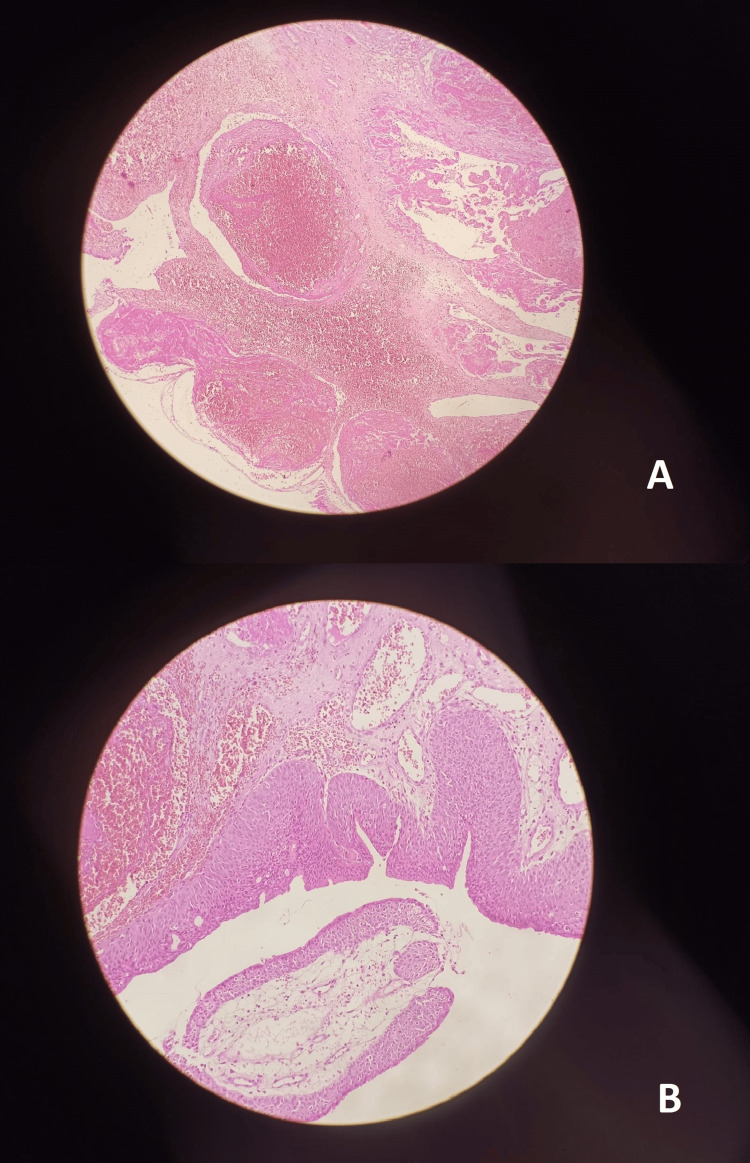
Histological appearance of the angiomatous urethral caruncle. A: Thrombosed ectatic vessels. B: Hyperplastic urethra.

Histopathological analysis showed an angiomatous urethral caruncle with no evidence of malignancy (Figure 3). At the one-month follow-up, the patient was asymptomatic with a completely healed urethral meatus.

## Discussion

First described by Samuel Sharp, urethral caruncle is a benign lesion that is relatively frequent in postmenopausal women [1]. Its occurrence in young women remains exceptional, while it is rare in men [2].

Macroscopically, it presents as a sessile polypoid lesion with a hemorrhagic surface and a crumbly consistency. Contrary to circumferential urethral prolapse, the base of the lesion is generally located on the mucosa of the posterior lip of the distal urethra. Histologically, it is lined with hyperplastic epithelium with clusters of capillaries in an inflammatory stroma [3]. In our case, this appearance was associated with an angiomatous variety characterized by thrombosed ectatic vessels.

The exact cause of urethral caruncle is unclear, and several nosological hypotheses have been put forward, including [4] (1) estrogen deficiency leading to a lack of attachment of the internal and external smooth muscle fibers of the urethra, resulting in protrusion of the mucosa. (2) Repeated infections and local trauma. (3) Intra-abdominal hyperpressure in the event of a hemorrhoidal crisis, for example.

All these theories suggest that irritation of the urethra is often associated with the presence of hemorrhagic and inflammatory plaques on pathological examination. In our case, trauma of the urethra during the use of endoscopic equipment, as well as contact of the mucosa with fragments of the calculus, could be incriminated as an etiological factor.

The clinical manifestations are dominated by the following symptoms: vaginal or even perineal pain (37%), hemorrhage in the form of metrorrhagia or haematuria (27%), and dysuria (20%) [5]. There are many differential diagnoses of varying frequency and severity, including [6] diverticulum of the urethra; prolapse of the urethra; leiomyoma and hemangioma (benign tumors); and leiomyosarcoma, primary carcinoma of the urethra, melanoma, and lymphoma (malignant tumors).

In the case of an atypical or suspicious mass, high-frequency vaginal ultrasound, CT, and MRI can be performed to better characterize the lesion and assess its stage of locoregional extension, while urethrocystoscopy can be used to look for intravesical invasion and obtain biopsies [7].

The first-line treatment is medical based on local estrogens, venotonics, and anti-inflammatories [8]. In the second line, after the failure of these drugs, surgical excision is necessary. Various techniques have been described, which are similar to those used for urethral prolapse (circumferential excision of the urethral mucosa, total excision using a bladder catheter, ligature at the base of the caruncle, fulguration, and cryotherapy) [9]. After surgical treatment, the literature shows a low recurrence rate of around 12% [10].

## Conclusions

Acquired angiomatous urethral caruncle is a rare complication after ureteroscopy, particularly in postmenopausal women. Information about the benign nature of this pathological entity helps to reduce patient anxiety, which, in turn, improves treatment outcomes.
